# Human Exposure to Hantaviruses Associated with Rodents of the *Murinae* Subfamily, Madagascar

**DOI:** 10.3201/eid2603.190320

**Published:** 2020-03

**Authors:** Harinirina Aina Rabemananjara, Vololoniaina Raharinosy, Ravo Michèle Razafimahefa, Jean Pierre Ravalohery, Jean Théophile Rafisandratantsoa, Soa Fy Andriamandimby, Minoarisoa Rajerison, Soanandrasana Rahelinirina, Aina Harimanana, Judickaelle Irinantenaina, Marie-Marie Olive, Christophe Rogier, Noël Tordo, Rainer G. Ulrich, Jean-Marc Reynes, Stéphane Petres, Jean-Michel Heraud, Sandra Telfer, Claudia Filippone

**Affiliations:** Institut Pasteur de Madagascar, Antananarivo, Madagascar (H.A. Rabemananjara, V. Raharinosy, R.M. Razafimahefa, J.P. Ravalohery, J.T. Rafisandratantsoa, S.F. Andriamandimby, M. Rajerison, S. Rahelinirina, A. Harimanana, J. Irinantenaina, M.-M. Olive, C. Rogier, J.-M. Heraud, C. Filippone);; Central Directorate of the French Military Health Service, Paris, France (C. Rogier); Institut Pasteur, Conakry, Guinea (N. Tordo);; Institut Pasteur, Paris (N. Tordo, J.-M. Reynes, S. Petres);; Federal Research Institute for Animal Health, Greifswald-Insel Riems, Germany (R.G. Ulrich);; German Center for Infection Research, Hamburg-Luebeck-Borstel-Insel Riems, Germany (R.G. Ulrich);; University of Aberdeen, Aberdeen, Scotland, UK (S. Telfer)

**Keywords:** hantavirus, Madagascar, seroprevalence, human population, rodents, viruses, zoonoses, Murinae

## Abstract

We conducted a national human serologic study of a hantavirus detected in Madagascar rodents using a commercial kit and a new ELISA targeting the virus. Our results suggest a conservative estimate of 2.7% (46/1,680) IgG seroprevalence. A second single-district study using the new ELISA revealed a higher prevalence (7.2%; 10/139).

Hantaviruses belonging to the genus *Orthohantavirus*, family *Hantaviridae*, are frequently zoonotic. Rodents are the usual reservoirs of human pathogenic hantaviruses and typically do not show obvious signs of disease (*1*,*2*). Transmission to humans usually occurs by inhalation of aerosols contaminated with urine or feces of infected reservoir animals (*3*). Hantaviruses are responsible for the severe illness hemorrhagic fever with renal syndrome (HFRS) and a milder form, nephropathia epidemica (NE), as well as for hantavirus cardiopulmonary syndrome (HCPS) (*1*).

Recent studies have described the geographic distribution and host range of novel hantaviruses in Africa and the Indian Ocean (*4**–**6*). In Madagascar, hantavirus RNA was identified by molecular analysis in *Rattus rattus* and *Eliurus majori* rats from a forest site in Anjozorobe district. The virus was named Anjozorobe virus (ANJZV) and is a genetic variant of Thailand orthohantavirus (THAIV) (*5*). In a more recent national study, Raharinosy et al. detected hantavirus RNA in 12% (n = 897) of *R. rattus* rats, and all the sequences obtained grouped with ANJZV (*7*), but they did not detect hantavirus RNA in *R. norvegicus* rats (0%; n = 124) (*7*), a species commonly associated with the cosmopolitan Seoul orthohantavirus (*1*). Because THAIV may cause HFRS in Southeast Asia (*8*), ANJZV could also be a human pathogen in Madagascar. In 1986, a limited study that used an immunofluorescence assay with Hantaan orthohantavirus (HTNV) and Puumala orthohantavirus antigens was conducted in areas around the capital and reported low titer hantavirus antibodies in the serum samples of 7/18 rat catchers in Madagascar (*9*).

We conducted a national study to assess hantavirus exposure in the general population of Madagascar. Sampling took place in conjunction with a recent rodent survey (*7*). In addition, because the original molecular hantavirus detection in Madagascar was from forest rodents (*5*), we also collected and analyzed human and rat samples from 4 sites close to forests.

## The Study

As part of a retrospective national study on zoonoses, we collected human serum samples from 2011–2013. We then randomly recruited 1,680 asymptomatic participants (851 female and 829 male; average age 37 years; range 18–99 years). We conducted sampling in 28 sites, each with urban and rural zones; we sampled 60 persons per site, with 30 persons per zone (*10*). In addition, we used samples collected during 2015–2016 from 4 rural sites close to natural forest areas in Moramanga district, which is close to Anjozorobe district. For this study, we randomly selected 139 asymptomatic participants (31–36 persons per site; average age 29 years, range 5–75 years). We also conducted trapping of the rat population in these 4 sites and randomly selected 237 *R. rattus* rats (58–61 per site).

The national ethics committee of Madagascar authorized human studies (authorization no. 066-MSANP/CE on July 26, 2011; no. 049-MSANT/CE on July 03, 2012). We conducted animal studies in accordance with Pasteur Institute animal use guidelines (https://www.pasteur.fr/en/file/2626/download?token=YgOq4QW7). A committee of the Institut Pasteur de Madagascar approved the studies.

For the national study, we performed initial screening using the commercial Dobrava-Hantaan IgG EIA kit (Reagena Ltd, https://www.reagena.com) based on the recombinant nucleocapsid (N) protein from HTNV. HTNV and THAIV, along with other *Murinae*-associated hantaviruses (Appendix Table 1), exhibit close antigenic relationship (*11*). However, because 2-way cross-reactivity is not complete (*12*), we developed a new IgG ELISA based on ANJZV recombinant N protein produced by a baculovirus-mediated insect cell expression system. We used this assay to test all samples testing positive or borderline by the commercial kit and a subset of negative samples (Appendix). Based on the apparent increased detection ability of the ANJZV ELISA, we only used ANJZV ELISA for testing the human samples from the 4 sites close to forest areas.

After screening 1,680 serum samples with the commercial ELISA, we found 36 (2.1%) positive and 26 (1.5%) borderline samples. Using the custom ANJZV ELISA on these samples and a subset of 62 negative samples, we found 46 positive and 15 borderline (Appendix Tables 2, 3). Thus, the ELISA we developed specifically for ANJZV appeared to be more sensitive. To obtain a conservative estimate of seroprevalence, only samples testing positive by both assays or positive by 1 assay and borderline by the other were considered positive; testing yielded an overall prevalence of 2.7% (46/1,680; 95% CI 2.0%–3.7%) in the population; 30 male (1.8%) and 16 female (0.9%) participants tested positive. 

Seropositive participants came from 20 of the 28 study sites (0–13.3% per site) distributed all over Madagascar (Table 1; Figure). Univariate generalized linear mixed models with site-zone as random effect indicated no effect of age, sex, or location (urban or rural), but we did find a slight suggestion of increased exposure in sites where our previous study (*7*) had detected infected rats (OR 3.0, 95% CI 0.78–11.5; p = 0.11).

**Table 1:** Seroprevalence of hantavirus in humans in the 28 sites used for national study, Madagascar*

Site no.	Site	No. positive/total no. participants (%; 95% CI)
1	Antananarivo	8/60 (13.3; 6.3–25.1)
2	Antsirabe	0/60 (0.0; 0.0–7.5)
3	Anjozorobe	1/60 (1.7; 0.0–10.1)
4	Tsiroanomandidy	3/60 (5.0; 1.3–14.8)
5	Antsiranana	0/60 (0.0; 0.0–7.5)
6	Sambava	2/60 (3.3; 0.5–12.5)
7	Nosy-be	2/60 (3.3; 0.5–12.5)
8	Mananjary	1/60 (1.7; 0.0–10.1)
9	Ambositra	3/60 (5.0; 1.3–14.8)
10	Farafangana	4/60 (6.7; 2.1–17.0)
11	Ihosy	0/60 (0.0; 0.0–7.5)
12	Fianarantsoa	1/60 (1.7; 0.0–10.1)
13	Antsohihy	1/60 (1.7; 0.0–10.1)
14	Mandritsara	0/60 (0.0; 0.0–7.5)
15	Maevatanana	4/60 (6.7; 2.1–17.0)
16	Ambato Boeny	0/60 (0.0; 0.0–7.5)
17	Mahajanga	2/60 (3.3; 0.5–12.5)
18	Moramanga	0/60 (0.0; 0.0–7.5)
19	Toamasina	2/60 (3.3; 0.5–12.5)
20	Ambatondrazaka	0/60 (0.0; 0.0–7.5)
21	Miandrivazo	1/60 (1.7; 0.0–10.1)
22	Ejeda	2/60 (3.3; 0.5–12.5)
23	Morombe	1/60 (1.7; 0.0–10.1)
24	Toliary	2/60 (3.3; 0.5–12.5)
25	Taolagnaro	2/60 (3.3; 0.5–12.5)
26	Ambovombe	2/60 (3.3; 0.5–12.5)
27	Belo sur Tsiribihina	0/60 (0.0; 0.0–7.5)
28	Morondava	2/60 (3.3; 0.5–12.5)
Total		46/1,680 (2.7; 2.0–3.7)

*Results from IgG testing by commercial ELISA and custom ELISA developed for Anjozorobe virus.

**Figure:**
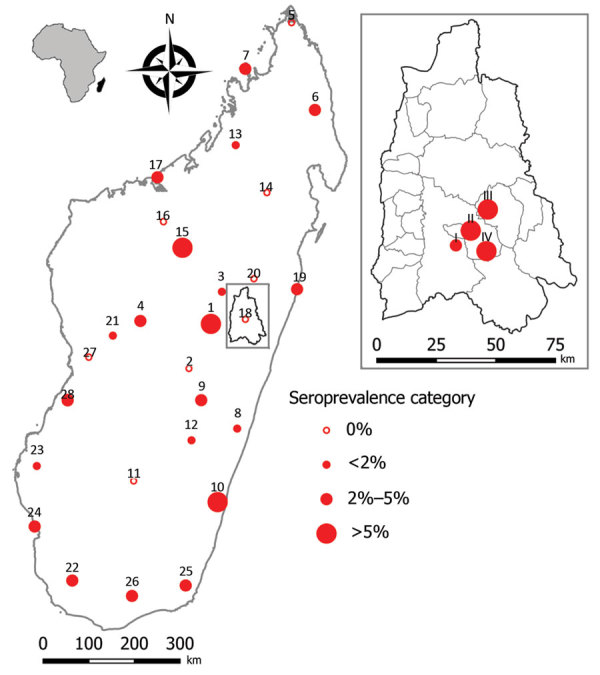
Geographic distribution of IgG hantavirus human seroprevalence in Madagascar for the 28 sites of the national-scale study and (inset) for the 4 sites close to forest in Moramanga district. Maps were built with QGIS software version 3.8.0—Zanzibar (Open Source Geospatial Foundation Project, http://qgis.osgeo.org). Small inset map shows location of Madagascar off the coast of Africa.

The Moramanga sites, situated close to forest, had significantly higher seroprevalence rates (7.2%: 10/139; 95% CI 3.7%–13.2%; range 3.2%–11.1%) than the national study sites (Kruskal-Wallis test χ^2^_1_ = 4.65; p = 0.03) (Table 2; Figure). This finding may partly reflect the apparent higher sensitivity of the ANJZV ELISA used for the regional study. Because 2 (n = 62, 3.2%) national samples tested negative by the commercial ELISA were positive by ANJZV ELISA, and 1,558 national samples were not tested by ANJZV ELISA, the overall national seroprevalence could be >2.7% (3.2% × 1,558 = 50; (50 + 46)/1,680 = 5.7%). Of interest, when we tested *R. rattus* rats from the 4 Moramanga sites by nested reverse transcription PCR using a protocol described previously (Appendix) (*7*), we also observed significantly higher infection rates than those for the national study sites; 77 of 237 rat samples were positive (32.5%; 95% CI 26.7%–38.9%; range 19.0%–43.3%; Kruskal-Wallis test χ^2^_1_ = 5.55; p = 0.02). These results further confirm a relatively high infection rate in the most abundant and widespread rodent in Madagascar. The small number of samples (2/61; 3%) negative by ANJZV ELISA but seropositive by the commercial ELISA could be explained by other *Murinae*-associated hantaviruses circulating in Madagascar.

**Table 2:** Seroprevalence of hantavirus in humans in the 4 sites close to forest in Moramanga district, Madagascar

Site no.	Site	No. positive/total no. participants (%; 95% CI)
I	Mangidifoza	1/31 (3.2; 0.1–18.5)
II	Atsahatsaka	4/36 (11.1; 3.6–27.0)
III	Sahamalotra	3/36 (8.3; 2.1–23.6)
IV	Ambalafary	2/36 (5.5; 1.0–20.0)
Total		10/139 (7.2; 3.7–13.2)

*Results from IgG testing by custom ELISA developed for Anjozorobe virus.

## Conclusions

Our results suggest the population of Madagascar is exposed to hantaviruses associated with the *Murinae* subfamily of rodents. The overall conservative prevalence estimate of 2.7% from the national-scale study, obtained using 2 ELISA assays, is similar to results from studies in some Africa countries where other confirmatory tests were used (3.9% in Cote d’Ivoire and 2.4% in the Democratic Republic of the Congo) (*4**,**13*). Although we believe some seropositive persons may have been exposed to other *Murinae*-associated hantaviruses, considering both ELISA results in humans and rodent infection data together (*7*), our observations are consistent with evidence that most were exposed to ANJZV. Specifically, the ANJZV ELISA detected more seropositive persons than the commercial kit, and the cosmopolitan Seoul virus, if present in rodents in Madagascar, is at low prevalence or patchily distributed (*7*). Because hantavirus infection rates in *R. rattus* rats appear higher at sites close to forest, more widespread testing with the ELISA developed for Anjozorobe virus is needed to confirm whether human communities in such areas are also at higher risk for infection. In addition, hospital surveillance studies are needed in Madagascar to determine if hantavirus infection occurs in patients, with testing focused on those with fever with unknown etiology, renal failure, or both.

Appendix Additional information about human exposure to hantaviruses associated with rodents of the *Murinae* subfamily, Madagascar
