# Supplementary material for: Human Exposure to Hantaviruses Associated with Rodents of the Murinae Subfamily, Madagascar
Source: Emerg Infect Dis. 2020 Mar;26(3):587–90. doi: 10.3201/eid2603.190320 (PMC7045827; doi:10.3201/eid2603.190320)
Supplement: Appendix — Additional information about human exposure to hantaviruses associated with rodents of the Murinae subfamily, Madagascar [file 19-0320-Techapp-s1.pdf]

Article DOI: https://doi.org/10.3201/eid2603.190320

Human Exposure to Hantaviruses Associated with Rodents of the *Murinae* Subfamily, Madagascar

Appendix

Commercial ELISA

We used Dobrava-Hantaan IgG enzyme immunoassay (Reagena Ltd, https://www.reagena.com) for initial screening of samples from a national-scale study in Madagascar. We chose this kit because it is a broad-spectrum assay based on recombinant nucleocapsid (N) protein from Hantaan virus (HTNV), a *Murinae*-associated hantavirus related to Anjozorobe virus (ANJZV).

Anjozorobe IgG ELISA

We developed an indirect IgG ELISA based on the use of the recombinant N protein of ANJZV. For the national study, we used this assay on all the samples testing positive or borderline by the commercial test, as well as on a subset of 62 samples tested negative by the commercial test. We selected the negative samples as matching controls (based on geographic site/zone, sex, and age) for each of the samples that were positive or borderline in the commercial assay.

Antigen Production

Sequence coding for the N protein (429 aa) of hantavirus strain Anjozorobe/Rr/MDG/2009/ATD56 (KC490916.1), flanked at N-ter by a KOZAK sequence and at C-ter by a sequence coding for an Enterokinase site (DDKC), followed by a sequence coding for a purification tag (Strep III) WSHPQFEKGGGSGGGSGGGSWSHPQFEK, was synthesized and inserted into the plasmid pVL1393 (Life Technologies SAS, https://www.thermofisher.com). This plasmid was cotransfected with linearized baculovirus DNA bestBac2.0 (Expressions Systems, expressionsystems.com) into *Spodoptera frugiperda* (Sf9) cells. We obtained first

generations of recombinant baculoviruses P1. We obtained the recombinant protein (antigen) used for our ELISA test through infection of Sf9 cells at MOI = 5 in a 5L wave bioreactor (GE Healthcare, https://www.gehealthcare.com) with the recombinant baculoviruses. We harvested the supernatant after 3 days of infection and then centrifuged at 6,000 rpm for 30 min. Supernatant was concentrated on AktaFlux and treated with avidin to remove biotin in the medium and with TRIS 1M to equilibrate the pH at 8. After a centrifugation of 20,000 rpm, we filtered supernatant with a 0,2 µm filter. We purified the result based on affinity streptag with an AKTA Avant system and Steptrap-HP 1mL column (GE Healthcare).

We confirmed protein presence and quantification by SDS PAGE gel, Western blot, and Bradford assay.

Homemade Anjozorobe IgG ELISA

We saturated wells of the microtiter plate with 100 µL of recombinant Anjozorobe antigen at the concentration of 5 µg/ml diluted in carbonate buffer (coated wells); in parallel, we saturated wells with 100 µL of carbonate buffer only (uncoated wells) (Appendix Figure). In both cases, we incubated the plate for 1 hour at 37°C. We then removed the buffer, and saturated the plate with PBS blocking buffer containing 0.05% Tween20 (PBS-T 0.05%) mixed with bovine albumin serum 1% (BSA 1%) and incubated for 1 hour at room temperature. We removed the buffer and washed the plate 3 times with PBS-T 0.1%, pH 7.2.

We added 100 µL of each serum diluted to 1/400 with PBS-T buffer 0.05% – BSA 0.5% and incubated for 1 hour at 37°C. Each serum was added in duplicate wells with (coated) and without (uncoated) antigen (Appendix Figure). We then washed the plate 3 times with PBS-T 0.1%, pH 7.2; 100 µL of IgG anti-human antibody, coupled to horseradish peroxidase, diluted at 1/6000 with PBS-T buffer 0.5% – BSA 0.5%, were added and incubated for 2 hours at 37°C. After washing, we added 100 µL of ABTS peroxidase substrate and incubated for 10 min in the dark. To stop the reaction, we added sulfuric acid and measured the optical density (OD) at wavelength of 450 nm.

Analysis

Samples where duplicates in coated wells or uncoated wells had a coefficient of variation >25% for OD were repeated. For each sample, we calculated the difference between the mean OD of coated wells and the mean OD of uncoated wells (ΔOD). We defined the exposure status

of tested persons using the ratio method; this involves comparing results to negative controls, ideally incorporating controls from the study population. In the first ANJZV assay, we employed 3 negative controls (NC) (Appendix Figure), including 2 samples from healthy Malagasy participants available in our biobank and the commercial assay negative control. The threshold for each plate was mean ΔOD for NC + 3 × standard deviation of ΔOD for NC. We calculated the mean ΔOD for NC for each individual plate, and calculated the standard deviation of ΔOD for NC over all plates run during the same week to account for variability between NC. In addition, when we detected variation between plates in the defined thresholds, we retested a subset of samples for confirmation. This included all borderline samples, as well as a subset of negative and positive samples. For these repeat assays, we included 5 negative controls obtained from healthy Malagasy participants, as well as the commercial test negative control; and a positive control corresponding to a serum sample that had consistently tested positive using the commercial test. We calculated the threshold for each plate using the ratio method as described above, but using the 5 Madagascar negative controls.

RT-PCR on *Rattus rattus* Samples

We tested RNA extracted from liver and spleen samples by nested RT-PCR using our established protocol *(1)*, which is based on a nested RT-PCR *(2)*. All positive samples were further confirmed using a recently developed real time RT-PCR assay based on a Taqman specific probe targeting the S sequence of hantaviruses of the Thailand group, which has been shown to have 100% specificity *(3)*. In all cases, RNAs positive by the nested RT-PCR were positive by the Taqman real-time RT-PCR, thus indicating specificity of such results.

References

1. Raharinosy V, Olive MM, Andriamiarimanana FM, Andriamandimby SF, Ravalohery JP, Andriamamonjy S, et al. Geographical distribution and relative risk of Anjozorobe virus (Thailand orthohantavirus) infection in black rats (*Rattus rattus*) in Madagascar. Virol J. 2018;15:83. PubMed https://doi.org/10.1186/s12985-018-0992-9

2. Klempa B, Fichet-Calvet E, Lecompte E, Auste B, Aniskin V, Meisel H, et al. Hantavirus in African wood mouse, Guinea. Emerg Infect Dis. 2006;12:838–40. PubMed https://doi.org/10.3201/eid1205.051487

3. Raharinosy V, Heraud JM, Rabemananjara HA, Telfer S, Rakoto DAD, Filippone C, et al. Fast, sensitive and specific detection of Thailand orthohantavirus and its variants using one-step real-time reverse-transcription polymerase chain reaction assay. Viruses. 2019;11:718. 10.3390/v11080718 PubMed https://doi.org/10.3390/v11080718

Appendix Table 1. Amino acid sequence similarities of the nucleocapsid protein of hantaviruses, Madagascar

Virus	HTNV	DOBV	ANJZV	THAIV
HTNV	–	83%	85%	84%
DOBV	83%	–	83%	83%
ANJZV	85%	83%	–	97%
THAIV	84%	83%	97%	–

*ANJZV, Anjozorobe virus variant of THAIV (accession no. YP_009362283.1); DOBV, Dobrava-Belgrade orthohantavirus (accession no. AES92931.1); HTNV, Hantaan orthohantavirus (accession no. ANK77968.1); THAIV, Thailand orthohantavirus (accession no. CAL37107.1).

Appendix Table 2. Comparison of results obtained during serologic analyses using a commercial IgG hantavirus kit and a custom Anjozorobe hantavirus ELISA on the same samples*

	ANJZV			
Commercial	Negative	Borderline	Positive	Total
Negative	56	4	2	62
Borderline†	3	9	**12**	24
Positive	2	**2**	**32**	36
Total	61	15	46	122

*Bold text indicates samples testing positive by both assays or borderline by one assay and positive by the other.
†Two additional samples tested borderline by commercial assay were not available for testing by the ANJZV IgG ELISA.

Appendix Table 3. Serologic results for samples detected as positive or borderline for hantavirus by commercial hantavirus ELISA kit (Reagena) and custom-developed Anjozorobe hantavirus ELISA, Madagascar*

		Case				Control			
Site no.	Site	Sex	Age, y	Reagena	ANJZV	Sex	Age, y	Reagena	ANJZV
1	Antananarivo	M	29	Positive	Positive	M	30	Negative	Negative
1	Antananarivo	M	39	Positive	Positive	M	37	Negative	Borderline
1	Antananarivo	F	23	Positive	Positive	F	23	Negative	Negative
1	Antananarivo	F	42	Positive	Positive	F	42	Negative	Negative
3	Anjozorobe	M	18	Positive	Positive	M	21	Negative	Negative
4	Tsiroanomandidy	M	22	Positive	Positive	M	21	Negative	Negative
4	Tsiroanomandidy	F	23	Positive	Borderline	F	23	Negative	Negative
6	Sambava	F	35	Positive	Negative	M	35	Negative	Negative
6	Sambava	M	45	Positive	Positive	M	46	Negative	Negative
7	Nosy-be	M	60	Positive	Positive	M	58	Negative	Negative
7	Nosy-be	F	57	Positive	Positive	F	58	Negative	Negative
8	Mananjary	M	48	Positive	Positive	M	46	Negative	Negative
9	Ambositra	F	70	Positive	Positive	M	61	Negative	Negative
9	Ambositra	M	33	Positive	Positive	M	32	Negative	Negative
9	Ambositra	M	41	Positive	Positive	M	41	Negative	Negative
10	Farafangana	M	67	Positive	Positive	M	65	Negative	Negative
12	Fianarantsoa	F	37	Positive	Positive	F	35	Negative	Negative
13	Antsohihy	M	39	Positive	Positive	M	40	Negative	Negative
15	Maevatanana	F	33	Positive	Negative	F	33	Negative	Positive
15	Maevatanana	F	23	Positive	Positive	M	23	Negative	Negative
17	Mahajanga	F	40	Positive	Positive	F	40	Negative	Negative
19	Toamasina	M	48	Positive	Positive	M	44	Negative	Negative
19	Toamasina	M	40	Positive	Positive	M	38	Negative	Negative
21	Miandrivazo	F	53	Positive	Positive	F	56	Negative	Negative
22	Ejeda	F	24	Positive	Positive	F	23	Negative	Negative
24	Toliary	M	54	Positive	Positive	M	57	Negative	Negative
24	Toliary	M	19	Positive	Positive	M	21	Negative	Negative
25	Taolagnaro	F	22	Positive	Positive	F	22	Negative	Negative
26	Ambovombe	M	44	Positive	Positive	M	45	Negative	Negative
27	Belo sur Tsiribihina	F	27	Positive	Borderline	F	24	Negative	Negative
28	Morondava	M	20	Positive	Positive	M	20	Negative	Borderline
1	Antananarivo	M	65	Borderline	Positive	M	80	Negative	Negative
1	Antananarivo	F	36	Borderline	Positive	F	35	Negative	Positive
1	Antananarivo	F	47	Borderline	Positive	F	47	Negative	Negative
4	Tsiroanomandidy	M	38	Borderline	Positive	M	42	Negative	Positive
5	Antsiranana	M	27	Borderline	Negative	M	25	Negative	Negative
6	Sambava	M	52	Borderline	Positive	M	65	Negative	Negative
9	Ambositra	M	26	Borderline	Borderline	M	26	Negative	Negative
10	Farafangana	F	38	Borderline	Positive	F	37	Negative	Negative
10	Farafangana	M	27	Borderline	Positive	M	29	Negative	Positive
10	Farafangana	M	22	Borderline	Positive	M	21	Negative	Negative
11	Ihosy	M	21	Borderline	NA	M	22	Negative	Negative
12	Fianarantsoa	M	35	Borderline	Negative	M	31	Negative	Negative
13	Antsohihy	M	34	Borderline	NA	M	36	Negative	Negative
15	Maevatanana	M	65	Borderline	Borderline	M	62	Negative	Negative
15	Maevatanana	M	61	Borderline	Positive	M	60	Negative	Negative
15	Maevatanana	F	38	Borderline	Positive	F	41	Negative	Positive
15	Maevatanana	M	25	Borderline	Positive	F	24	Negative	Negative
16	Ambato Boeny	M	100	Borderline	Negative	M	69	Negative	Negative
16	Ambato Boeny	F	18	Borderline	Borderline	F	20	Negative	Negative
17	Mahajanga	F	43	Borderline	Positive	F	43	Negative	Negative
19	Toamasina	M	56	Borderline	Negative	M	43	Negative	Negative
20	Ambatondrazaka	F	23	Borderline	Borderline	F	23	Negative	Negative
22	Ejeda	M	33	Borderline	Positive	M	31	Negative	Negative
23	Morombe	M	45	Borderline	Negative	M	44	Negative	Negative
23	Morombe	M	27	Borderline	Positive	M	27	Negative	Negative
23	Morombe	F	24	Borderline	Borderline	F	23	Negative	Negative
24	Toliary	F	30	Borderline	Borderline	F	31	Negative	Negative
25	Taolagnaro	M	26	Borderline	Positive	M	30	Negative	Negative
26	Ambovombe	M	76	Borderline	Positive	M	62	Negative	Negative
27	Belo sur Tsiribihina	M	28	Borderline	Borderline	M	29	Negative	Negative
28	Morondava	M	18	Borderline	Positive	M	18	Negative	Negative

*Samples testing negative by commercial ELISA matched by site/zone, sex, and age. ANJZV, Anjozorobe orthohantavirus ELISA; NA, not available.

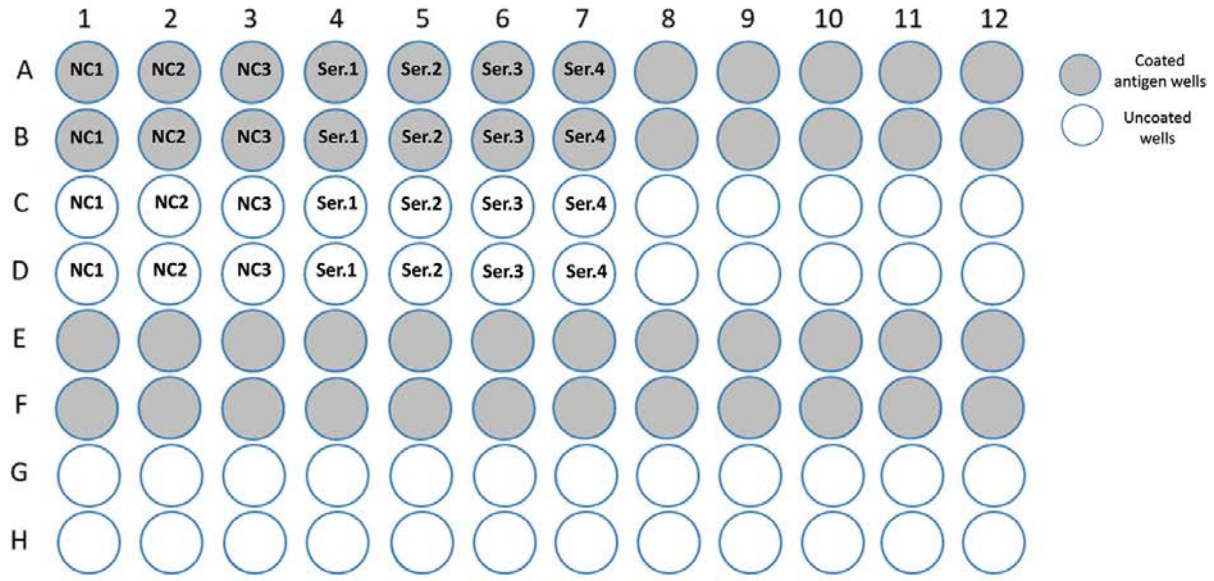

Appendix Figure. Schematic plan of plate used in Anjozorobe hantavirus ELISA. Gray circles indicate wells containing recombinant Anjozorobe antigen. White circles indicate wells with buffer solution only. Ser., serum.
